# Crude Preparations of Helicobacter pylori Outer Membrane Vesicles Induce Upregulation of Heme Oxygenase-1 via Activating Akt-Nrf2 and mTOR–IκB Kinase–NF-κB Pathways in Dendritic Cells

**DOI:** 10.1128/IAI.00190-16

**Published:** 2016-07-21

**Authors:** Su Hyuk Ko, Da Jeong Rho, Jong Ik Jeon, Young-Jeon Kim, Hyun Ae Woo, Nayoung Kim, Jung Mogg Kim

**Affiliations:** a Department of Microbiology and Department of Biomedical Science, Hanyang University College of Medicine and Graduate School of Biomedical Science and Engineering, Seoul, South Korea; b Department of Biotechnology, Joongbu University, Gumsan, South Korea; c Graduate School of Pharmaceutical Sciences, Ewha Womans University, Seoul, South Korea; d Department of Internal Medicine, Seoul National University Bundang Hospital, Seongnam, South Korea; University of Illinois Urbana

## Abstract

Helicobacter pylori sheds outer membrane vesicles (OMVs) that contain many surface elements of bacteria. Dendritic cells (DCs) play a major role in directing the nature of adaptive immune responses against H. pylori, and heme oxygenase-1 (HO-1) has been implicated in regulating function of DCs. In addition, HO-1 is important for adaptive immunity and the stress response. Although H. pylori-derived OMVs may contribute to the pathogenesis of H. pylori infection, responses of DCs to OMVs have not been elucidated. In the present study, we investigated the role of H. pylori-derived crude OMVs in modulating the expression of HO-1 in DCs. Exposure of DCs to crude H. pylori OMVs upregulated HO-1 expression. Crude OMVs obtained from a *cagA*-negative isogenic mutant strain induced less HO-1 expression than OMVs obtained from a wild-type strain. Crude H. pylori OMVs activated signals of transcription factors such as NF-κB, AP-1, and Nrf2. Suppression of NF-κB or Nrf2 resulted in significant attenuation of crude OMV-induced HO-1 expression. Crude OMVs increased the phosphorylation of Akt and downstream target molecules of mammalian target of rapamycin (mTOR), such as S6 kinase 1 (S6K1). Suppression of Akt resulted in inhibition of crude OMV-induced Nrf2-dependent HO-1 expression. Furthermore, suppression of mTOR was associated with inhibition of IκB kinase (IKK), NF-κB, and HO-1 expression in crude OMV-exposed DCs. These results suggest that H. pylori-derived OMVs regulate HO-1 expression through two different pathways in DCs, Akt-Nrf2 and mTOR–IKK–NF-κB signaling. Following this induction, increased HO-1 expression in DCs may modulate inflammatory responses in H. pylori infection.

## INTRODUCTION

The gastric pathogen Helicobacter pylori is a causative agent of diseases such as chronic gastritis, peptic ulcers, gastric cancers, and gastric mucosa-associated lymphoid tissue (MALT) lymphoma. Although the bacteria do not directly invade the gastric lamina propria, the infection leads to infiltration of several immune cells (1). A murine model of H. pylori infection showed an influx of dendritic cells (DCs) into the paragastric lymph nodes that reached maximal levels at 21 days postinfection (2). In addition, many DCs are observed in human gastric mucosa infected with H. pylori (1). Interactions between the pathogen and DCs play a major role in directing the nature of the adaptive immune response against H. pylori (3, 4). DCs recognize pathogen-associated molecular patterns (PAMPs) present on H. pylori, after which their interactions trigger host cell signaling cascades, playing a critical role in the induction of immunity.

The majority of H. pylori in the stomach remains unattached to the surface epithelium even though the bacteria can adhere to gastric epithelial cells (5, 6). H. pylori bacteria release portions of their outer membrane in vesicular form; these compartments are referred to as outer membrane vesicles (OMVs) (7). Since OMVs are derived from the outer membrane of bacteria, they contain many surface elements of H. pylori, such as CagA, lipopolysaccharide (LPS), and outer membrane proteins (7–11). These vesicles also act as a vehicle for vacuolating cytotoxin (VacA) (12). The released H. pylori OMVs are internalized to gastric epithelial cells (6, 9, 12). After internalization, OMVs can modulate gastric epithelial cell proliferation, induce apoptosis, and stimulate secretion of interleukin-8 (IL-8) (9, 13). In addition, H. pylori OMVs can induce human eosinophil degranulation (5). Based on these results, H. pylori-derived OMVs may contribute to regulation of H. pylori-induced pathogenic effects that have been observed in the stomach.

PAMPs or several proinflammatory cytokines can induce expression of heme oxygenase-1 (HO-1) (14). HO-1 is an inducible enzyme that catalyzes the degradation of free heme into carbon monoxide, biliverdin, and free iron. Upregulated HO-1 expression leads to adaptive immune responses that protect cells from immunopathogenesis or stress damage (14, 15). With H. pylori infection, exposure of H. pylori to macrophage induced *hmox-1*, the gene encoding HO-1 (16). HO-1 expression was also increased in gastric mononuclear cells of human patients and macrophages of mice infected with cagA^+^ H. pylori strains (16). These findings enabled us to develop a hypothesis that the induction of HO-1 may regulate inflammatory responses induced by H. pylori-derived OMVs. Nevertheless, there have been no reports regarding OMV-induced HO-1 expression.

Signals from transcription factors, including nuclear factor-kappaB (NF-κB), activator protein-1 (AP-1), and NF-E2-related factor 2 (Nrf2, or nuclear factor [erythroid-derived 2]-like 2 [NFE2L2]), are known to regulate the expression of HO-1 (15). Stimulation of intestinal epithelial cells and DCs with H. pylori or H. pylori-derived materials can activate NF-κB and AP-1 signaling (17). These observations raise the possibility that signaling molecules may be activated in H. pylori-derived OMV-exposed DCs to regulate HO-1 expression. However, there is no evidence that the OMV-induced signaling pathway leads to HO-1 induction in DCs. In the present study, we investigated HO-1 induction in response to stimulation of DCs with H. pylori-derived crude OMVs. We found that two signaling pathways involving Akt-Nrf2 activation and mammalian target of rapamycin (mTOR)–IκBα kinase (IKK)–NF-κB activation are required for HO-1 induction following exposure of DCs to crude OMVs. These data suggest that increased HO-1 expression in DCs modulates inflammatory responses in H. pylori infection.

## MATERIALS AND METHODS

### Reagents.

LPS-free fetal bovine serum (FBS), antibiotics (10,000 units/ml penicillin, 10,000 μg/ml streptomycin, and 25 μg/ml amphotericin B), l-glutamine, TRIzol, and Ca^2+^- and Mg^2+^-free Hanks' balanced salt solution (HBSS) were all obtained from Gibco BRL (Gaithersburg, MD, USA). Everolimus, rapamycin, β-cyclodextrin, and RPMI 1640 medium were purchased from Sigma Chemical Co. (St. Louis, MO, USA). Monoclonal antibodies (MAbs) against phospho-IκBα, phospho-c-jun, phospho-p65, IKKα, IKKβ, phospho-IKKα/β, S6 kinase 1 (S6K1), phospho-S6K1, Akt1, phospho-Akt1, mTOR, and actin were acquired from Cell Signaling Technology, Inc. (Beverly, MA, USA). Anti-phospho-Nrf2 was purchased from Bioss Antibodies, Inc. (Woburn, MA, USA). Antibodies (Abs) against HO-1, Nrf2, p50, p52, p65, c-Rel, Rel B, c-Jun, c-Fos, JunB, JunD Fos-B, and lamin B and goat anti-mouse and anti-rabbit secondary Abs conjugated to horseradish peroxidase were acquired from Santa Cruz Biotechnology (Santa Cruz, CA, USA). Alexa Fluor 488 and DyLight 549 secondary Abs were purchased from Thermo Fisher Scientific (Waltham, MA, USA) and Abcam (Cambridge, MA, USA), respectively. Bay 11-7085, SR11302, MK-2206, and MHY1485 were obtained from Calbiochem (La Jolla, CA, USA), Tocris Bioscience (Bristol, United Kingdom), Selleckchem (Houston, TX, USA), and Millipore (Bedford, MA, USA), respectively. NF-κB essential modifier (NEMO)-binding domain (NBD) peptides were obtained from Peptron (Daejeon, South Korea) to block association of NEMO with the IKK complex and inhibit NF-κB activation. Sequences of wild-type and mutant peptides are DRQIKIWFQNRRMKWKKTALDWSWLQTE and DRQIKIWFQNRRMKWKKTALDASALQTE, respectively. Positions of the W → A mutations are underlined (18).

### H. pylori strains.

H. pylori strain 60190 (ATCC 49503; CagA^+^ *vacA* s1a/m1) was used to purify crude OMVs. The CagA^−^ isogenic mutant, VacA^−^ isogenic mutant, and PicB^−^/CagE^−^ isogenic mutant were obtained from Yong Chan Lee (Yonsei University College of Medicine, Seoul, South Korea) with the kind permission of Martin J. Blaser (New York University Langone Medical Center, NY, USA) (5). All H. pylori strains were cultured under microaerophilic conditions (5% O_2_, 10% CO_2_, and 85% N_2_).

### Preparation of crude H. pylori OMVs.

H. pylori OMVs were prepared according to a previously described protocol (5, 19). Briefly, H. pylori was grown in brucella broth supplemented with 0.6% (wt/vol) β-cyclodextrin at 37°C under microaerobic conditions with constant rotation (120 rpm). Two methods of preparation of OMVs were used in this study. After 72 h of growth the bacteria were pelleted by centrifugation (12,000 × *g*, 15 min, 4°C), and the resulting supernatant was then filtered through a 0.22-μm-pore-size filter. The final supernatants were ultracentrifuged (200,000 × *g*, 2 h, 4°C) to recover OMVs. After three washes in phosphate-buffered saline (PBS), crude OMVs were stored at −20°C until required. The protein concentrations of OMV preparations were determined by the Bradford method (Bio-Rad, Hercules, CA, USA). Medium without bacteria was used as a control.

### Generation of primary murine BM-derived and human monocyte-derived DCs.

Specific pathogen-free C57BL/6 and breeding pairs of Nrf2^−/−^ knockout mice were obtained from Orient Experimental Animals (Seoungnam, South Korea) and RIKEN BioResource Center (Tsukuba, Japan) (20), respectively. All animal experiments were performed according to protocols approved by the Institutional Animal Care and Use Committee of Hanyang University. Experiments using Nrf2^−/−^ knockout mice were approved by the Institutional Animal Care and Use Committee of Ewha Womans University. To generate bone marrow (BM)-derived DCs, femurs and tibias of male mice (8 to 12 weeks of age; body mass of 20 to 25 g) were harvested, and BM cells were obtained as previously described (17). Cells were then cultured in RPMI 1640 medium supplemented with 10% FBS, 1% antibiotics, l-glutamine (2 mM), 2-mercaptoethanol (55 μM), murine recombinant granulocyte-macrophage colony-stimulating factor (GM-CSF; 10 ng/ml) (PeproTech, Rocky Hill, NJ, USA), and murine recombinant IL-4 (10 ng/ml; PeproTech). After 6 days in culture, DCs were harvested and stimulated with crude OMVs. The purity of CD11c^+^ cells was greater than 95% as determined by flow cytometric analysis.

Human monocyte-derived DCs were generated as previously described (21, 22). Briefly, CD14^+^ cells were purified from peripheral blood mononuclear cells (PBMCs) using a magnetic cell separation system (Miltenyi Biotec, Bergisch-Gladbach, Germany). The purity of CD14^+^ cells counted using fluorescein isothiocyanate (FITC)-conjugated mouse anti-CD14 MAb and flow cytometry was >90%. To generate human DCs, purified CD14^+^ cells were plated in ultralow-adherence 24-well plates (Corning Costar, Sigma-Aldrich) at a density of 5 × 10^5^ cell/ml and cultured in RPMI 1640 medium supplemented with 10% FBS, 1% antibiotics, l-glutamine (2 mM), 2-mercaptoethanol (55 μM), human recombinant GM-CSF (50 ng/ml), and IL-4 (20 ng/ml). The study was approved by the Ethics Committee of Hanyang University.

DC2.4, an immature murine DC line, was cultured in RPMI 1640 medium supplemented with 10% FBS, 1% antibiotics, l-glutamine (2 mM), sodium pyruvate (1 mM), and nonessential amino acids (2 mM). These cells were grown at 37°C with 5% CO_2_ as previously described (17). Cells were seeded at 0.5 × 10^6^ to 2 × 10^6^ cells per well onto six-well plates and allowed to attach overnight. After 12 h of serum starvation, cells were incubated with OMVs.

### Quantitative RT-PCR.

Cells were treated with crude OMVs, after which total cellular RNA was extracted using TRIzol, and reverse transcription-PCR (RT-PCR) amplification was performed. The primers and expected PCR product sizes were as follows: mouse HO-1, 5′-AAG AGG CTA AGA CCG CCT TC-3′ (sense) and 5′-GTC GTG GTC AGT CAA CAT GG-3′ (antisense), yielding a 591-bp fragment (GenBank accession number NM_010442.2; Mus musculus heme oxygenase [decycling] 1 [Hmox1] mRNA) (23); mouse β-actin, 5′-GTG GGC CGC TCT AGG CAC CAA-3′ (sense) and 5′-CTC TTT GAT GTC ACG CAC GAT TTC-3′ (antisense), yielding a 540-bp fragment (GenBank accession number NM_007393.4; Mus musculus actin, beta [Actb] mRNA) (18). To quantify mRNA molecules, standard RNAs for mouse HO-1 and β-actin were generated by *in vitro* transcription using T7 RNA polymerase. The sizes of PCR products generated from standard RNAs for mouse HO-1 and β-actin are 478 bp and 746 bp, respectively.

### EMSAs.

Cells were harvested, and nuclear extracts were prepared as described previously (24). The concentration of protein in extracts was determined using the Bradford assay (Bio-Rad, Hercules). Electrophoretic mobility shift assays (EMSAs) were performed according to the manufacturer's instructions (Promega, Madison, WI, USA). In brief, 5 μg of nuclear extract was incubated for 30 min at room temperature with γ-^32^P-labeled oligonucleotide probes (5′-AGT TGA GGG GAC TTT CCC AGG C-3′ for the NF-κB binding site; 5′-CGC TTG ATG ACT CAG CCG GAA-3′ for the AP-1 binding site; 5′-TGG GGA ACC TGT GCT GAG TCA CTG GAG-3′ for the Nrf2 binding site). After incubation, both bound DNA and free DNA were resolved on 5% polyacrylamide gels, as described previously (24). Supershift assays were used to identify specific members of the NF-κB or AP-1 family activated by crude OMV stimulation. EMSAs were performed as described above, except that rabbit Abs (1 μg/reaction volume) against NF-κB proteins p50, p52, p65, c-Rel, or Rel B were added during the binding reaction period. For AP-1 supershift assays, rabbit Abs (1 μg/reaction volume) against c-Jun, c-Fos, JunB, JunD, or Fos-B were used. For Nrf2 supershift assays, anti-Nrf2 Ab (1 μg/reaction volume) and an IgG isotype control Ab were used. A competition assay for Nrf2 signals was performed by adding 100-fold excess of unlabeled probe (cold probe) prior to the addition of radiolabeled probe (hot probe) or mutant probe to the reaction mixture. The sequence of the mutant oligonucleotide was 5′-TGG GGA ACC TGT GCT AGG TCA CTG GAG-3′ (the mutation is underlined). Oligonucleotide probes for the NF-κB- or AP-1-binding assay were purchased from Promega, and oligonucleotides for the Nrf2 assay were obtained from Santa Cruz Biotechnology. Nuclear extracts obtained from DC2.4 cells treated with tumor necrosis factor alpha (TNF-α; 20 ng/ml) for 1 h were used as positive controls for NF-κB and AP-1. Nuclear extracts obtained from DC2.4 cells treated with curcumin (10 μg/ml) for 6 h were used as a positive control for Nrf2. Two negative controls were used: (i) no extracts and (ii) nuclear extracts obtained from DC2.4 cells transfected with lentivirus containing each dominant negative (lentivirus-dn) plasmid or short hairpin RNAs (shRNAs).

### Transfection assay.

Lentiviral systems containing mammalian expression vectors encoding a hemagglutinin (HA) epitope-tagged mutant IκBα (lentivirus–IκBα-AA) with substitutions of serines for alanines at positions 32 and 36 and an HA epitope-tagged mutant c-jun (TAM67) with deletions of amino acids at positions 3 to 122 were used to block NF-κB and AP-1 activation, respectively (18). Lentiviral vectors containing an *Nrf2* shRNA plasmid (mouse) or *IKKβ* shRNA plasmid (mouse) and control lentivirus were purchased from Santa Cruz Biotechnology. Transfection experiments were performed according to the manufacturer's instructions.

Small interfering RNAs (siRNAs) against the *p65* subunit of NF-κB complex, *c-jun* subunit of AP-1 complex, *Akt1*, and *mTOR* were designed as described previously (18). The siRNAs were synthesized by Qiagen (Valencia, CA, USA). A negative (nonsilencing) siRNA control (NS-RNA) was also purchased from Qiagen. Briefly, cells were cultured in six-well plates with 50% to 80% confluence. The cells were transfected with the siRNA using Fugene 6 (Roche, Mannheim, Germany) as a transfection reagent, as described previously (18). Transfected cells were incubated for 48 h prior to the assay.

### Immunoblotting and enzyme-linked immunosorbent assay (ELISA).

Cells were washed with ice-cold PBS and lysed in 0.5 ml/well lysis buffer (150 mM NaCl, 20 mM Tris, pH 7.5, 0.1% Triton X-100, 1 mM phenylmethylsulfonyl fluoride [PMSF], and 10 μg/ml aprotinin). Protein, at 15 to 50 μg per lane, was size fractionated on a polyacrylamide minigel (Mini-Protein II; Bio-Rad) and electrophoretically transferred to a nitrocellulose membrane (0.1-μm pore size). The immunoreactive proteins to which primary Abs bound were visualized using goat anti-rabbit or anti-mouse secondary Abs conjugated to horseradish peroxidase, followed by enhanced chemiluminescence (ECL system; Amersham Life Science, Buckinghamshire, England) and exposure to X-ray film (18).

The level of HO-1 proteins was measured using a commercially available kit (R&D Systems, Inc., Minneapolis, MN, USA). A PathScan phospho-IκBα kinase assay kit was obtained from Cell Signaling Technology. ELISA kits of the TransAM NF-κB family and TransAM Nrf2 were purchased from Active Motif (Carlsbad, CA, USA) (18). Each assay was performed according to each manufacturer's instructions.

### Immunofluorescence assay.

Cells were seeded (5 × 10^4^ cells in 0.2 ml of RPMI 1640 medium/well) on eight-well poly-d-lysine-coated culture microslides (Santa Cruz). After exposure to crude OMVs, cells were treated with 0.3% Triton X-100 in PBS for 30 min at room temperature, followed by incubation with goat anti-HO-1 and rabbit anti-phospho-p65 Abs as primary Abs for 2 h. In another experiment to evaluate HO-1 expression and phospho-Nrf2 translocation, cells were treated with goat anti-HO-1 and rabbit anti-phospho-Nrf2 Abs as primary Abs for 2 h. Cells were then treated with Alexa Fluor 488-conjugated secondary Ab (green) against goat IgG and DyLight 549-conjugated secondary Ab (red) against rabbit IgG for 1 h. Images were captured using a DMI4000B (Leica Microsystems GmbH, Wetzlar, Germany) fluorescence microscope (25).

### Statistical analyses.

Data from quantitative RT-PCR assays are presented as means ± standard deviations (SD), and ELISA data are presented as means ± standard errors of the means (SEM). A Mann-Whitney *t* test was used for statistical analysis. *P* values of <0.05 were considered statistically significant.

## RESULTS

### Crude H. pylori OMVs induce HO-1 expression in DCs.

**FIG 1:**
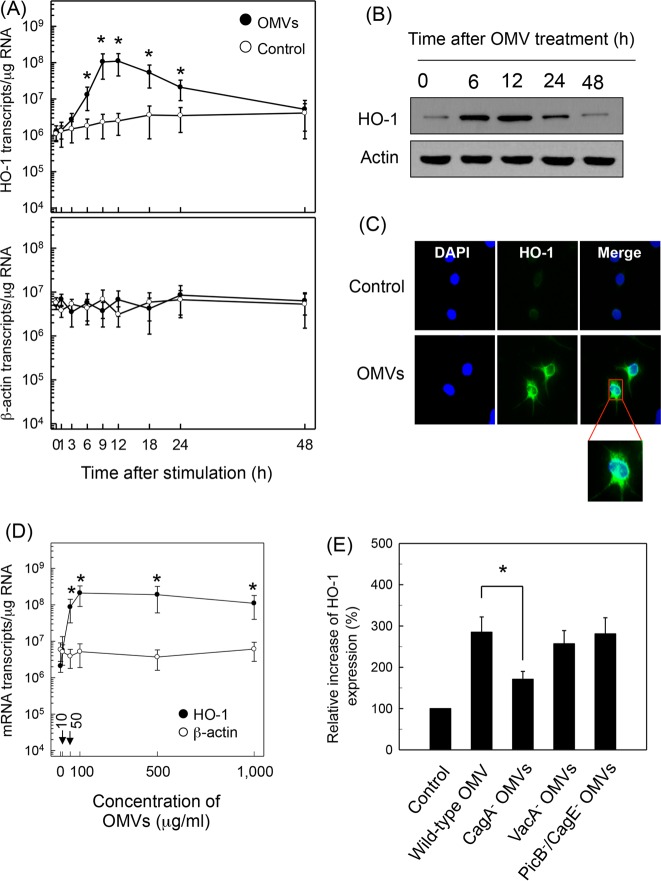
Upregulation of HO-1 in DCs treated with crude OMVs. (A) Time courses of HO-1 mRNA expression in murine BM-derived DCs after treatment with crude OMVs. DCs were treated with crude OMVs (50 μg/ml) for the indicated periods of time. Levels of HO-1 and β-actin mRNAs were analyzed by quantitative RT-PCR using each standard RNA. Values are expressed as means ± SD (*n* = 5). *, *P* < 0.05, for results compared to those with untreated control (0 h). (B) DCs were treated with OMVs (50 μg/ml) for the indicated period of time. Protein levels of HO-1 and actin were determined using immunoblot analysis. These results are a representative of three independent experiments. (C) DCs were incubated with or without crude OMVs (50 μg/ml) for 12 h. Cells were stained with anti-HO-1 (green) and 4′,6′-diamidino-2-phenylindole (DAPI; blue, nucleus) and were visualized with fluorescence microscopy (magnification, ×400). Results are representative of three independent experiments. (D) DCs were treated with indicated concentrations of crude OMVs for 9 h. Expression of HO-1 (filled circles) and β-actin (open circles) mRNAs was analyzed by quantitative RT-PCR using a standard RNA for each. Values are expressed as means ± SD (*n* = 5). *, *P* < 0.05, for results compared with those of the untreated control. (E) Murine BM-derived DCs were stimulated with crude OMVs (50 μg/ml) obtained from wild-type H. pylori or the indicated isogenic mutants for 12 h. Protein expression of HO-1 was measured using ELISA kits. Data are expressed as mean fold induction ± SEM (%) relative to that of the untreated controls (*n* = 5). *, *P* < 0.05.

Stimulation of murine BM-derived DCs with crude OMVs increased the expression of HO-1 mRNA transcripts, as assessed by quantitative RT-PCR. Increased HO-1 mRNA expression was first noted at 6 h after stimulation, with a peak observed at 9 to 12 h poststimulation. HO-1 expression gradually diminished to baseline thereafter (Fig. 1A). To verify that the expressed HO-1 transcripts are linked to protein synthesis, expression of HO-1 proteins was analyzed by immunoblot analysis. As shown in Fig. 1B, stimulation of DCs with crude OMVs resulted in increased expression of HO-1 proteins. Immunohistochemical analyses also showed that the expression of HO-1 molecules in OMV-exposed DCs was higher than that of unstimulated cells (Fig. 1C). The magnitude of HO-1 expression was dependent on the concentration of crude OMVs used for stimulation (Fig. 1D). The concentration of crude OMVs that gave a half-maximal response (50% effective concentration [EC_50_]) was 50 μg/ml, as calculated by SigmaPlot, version 10.0, software (Systat Software Inc., San Jose, CA, USA). Based on these results, 50 μg/ml of crude OMVs was used in subsequent experiments.

OMVs are known to contain many surface elements and bacterial proteins, including CagA, VacA, and LPS (7–11). To investigate the role of H. pylori virulence factors on HO-1 expression, crude OMVs obtained from H. pylori mutant strains were added to BM-derived DCs. Results showed that a CagA-negative isogenic mutant strain induced less HO-1 expression than crude OMVs obtained from a wild-type strain. However, HO-1 expression by crude OMVs obtained from a VacA^−^ or a PicB^−^/CagE^−^ isogenic mutant was similar to that obtained with a wild-type strain (Fig. 1E).

### Activation of NF-κB is essential to upregulate HO-1 expression in crude H. pylori OMV-exposed DCs.

**FIG 2:**
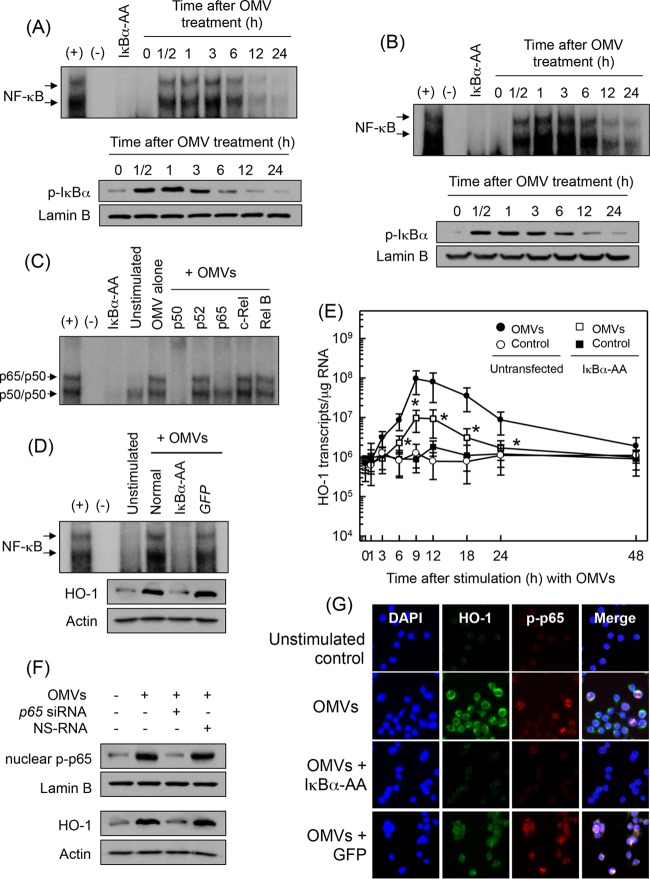
Effects of NF-κB suppression on HO-1 expression in DCs treated with crude OMVs. (A and B) Activation of NF-κB in DCs stimulated with crude OMVs. BM-derived DCs (A) and DC2.4 cells (B) were treated with crude OMVs (50 μg/ml) for the indicated times. NF-κB DNA binding activity was assessed by EMSA. Immunoblot results for concurrent phospho-IκBα and lamin B in nuclear extracts under the same conditions are provided beneath the EMSA panels. Results are representative of more than three independent experiments. (C) Supershift assays using nuclear extracts from BM-derived DCs treated with crude OMVs (50 μg/ml) for 1 h were performed using Abs against p50, p52, p65, c-Rel, and Rel B. +, positive control; −, no extracts; IκBα-AA, nuclear extracts obtained from lentivirus containing IκBα superrepressor-transfected DC2.4 cells stimulated with crude OMVs (50 μg/ml) for 1 h. (D) DC2.4 cells were transfected with either lentivirus containing IκBα-superrepressor (IκBα-AA) or control virus (*GFP*). Transfected cells were stimulated with crude OMVs (50 μg/ml) for 1 h. NF-κB binding activity was assayed by EMSA (top panel). Transfected or untransfected cells were treated with crude OMVs (50 μg/ml) for 12 h. Expression of HO-1 and actin proteins was analyzed by immunoblotting (bottom panels). Results are representative of more than three independent experiments. (E) Transfected DC2.4 cells were treated with crude OMVs (50 μg/ml) for the indicated periods of time. Levels of HO-1 mRNA were analyzed by quantitative RT-PCR using a standard RNA. Values are expressed as means ± SD (*n* = 5). β-Actin mRNA levels in each group remained relatively constant throughout the same periods (∼ 10^6^ transcripts/μg total RNA). *, *P* < 0.05, for results compared with those in untransfected cells treated with crude OMVs. (F) DC2.4 cells were transfected with NF-κB *p65*-specific siRNA or a nonsilencing siRNA (NS-RNA) as a control for 48 h, after which cells were combined with crude OMVs (50 μg/ml) for 1 h. Nuclear extracts were analyzed by immunoblotting with the indicated Abs (top panels). Transfected cells were stimulated with crude OMVs (50 μg/ml) for 12 h. Expression of HO-1 and actin proteins was analyzed by immunoblotting (bottom panels). Results shown are representative of more than three independent experiments. (G) DC2.4 cells were transfected with IκBα-AA or control virus (*GFP*). Transfected cells were stimulated with crude OMVs (50 μg/ml) for 6 h, and immunofluorescence microscopy was performed. Cells were stained with anti-HO-1 Ab (green), anti-phospho-p65 Ab (red), and 4′,6′-diamidino-2-phenylindole (DAPI; blue, nucleus). Data are representative of at least five experiments.

The promoter region of the HO-1 gene contains binding sites for NF-κB and AP-1. Although stimulation of gastric epithelial cells with H. pylori OMVs upregulates NF-κB (9), there is no report on whether H. pylori OMVs activate transcription factors in DCs. Therefore, we first examined whether crude OMV exposure could activate the signals of NF-κB or AP-1 in DCs. As shown in Fig. 2A, stimulation of BM-derived DCs with crude OMVs increased NF-κB DNA binding, as assessed by EMSA. In addition, phosphorylated IκBα was detected in crude OMV-exposed DCs. Similar results were observed in DC2.4 cells stimulated with OMVs (Fig. 2B). To identify the specific NF-κB subunits comprising the NF-κB signal detected by EMSAs in crude OMV-stimulated DCs, supershift assays were performed. As shown in Fig. 2C, Abs to p65 and p50 shifted the NF-κB signal significantly. In contrast, the anti-p52, anti-c-Rel, and anti-Rel B Abs did not shift the NF-κB signal.

To evaluate whether OMV-induced NF-κB activation might be associated with HO-1 expression in crude DCs, transfection with lentivirus–IκBα-AA was used. Transfected DC2.4 cells were stimulated with crude OMVs for 1 h, and the NF-κB DNA binding activity was assessed by EMSA. Transfection with lentivirus–IκBα-AA suppressed NF-κB activity to control levels in crude OMV-treated cells. However, control lentivirus containing a green fluorescent protein (GFP)-expressing plasmid did not reduce NF-κB activation (Fig. 2D). Concurrently, the expression of HO-1 proteins induced by crude OMVs was definitely affected when NF-κB activity was suppressed. Consistent with these results, transfection with lentivirus–IκBα-AA significantly decreased HO-1 mRNA expression in DC2.4 cells under crude OMV-stimulated conditions (Fig. 2E). To confirm these results, another experiment was performed using *p65* siRNA to suppress NF-κB activity. The *p65* siRNA almost completely suppressed nuclear phospho-p65 activity in OMV-exposed DC2.4 cells (Fig. 2F). In this experimental system, blocking NF-κB with *p65* siRNA apparently suppressed the crude OMV-induced increase of HO-1 protein expression in DC2.4 cells (Fig. 2F). Moreover, immunofluorescence microscopy showed that expression of phospho-p65 and HO-1 increased in crude OMV-exposed DC2.4 cells. In contrast, cells transfected with lentivirus–IκBα-AA definitely suppressed phospho-p65 and HO-1 protein expression (Fig. 2G).

**FIG 3:**
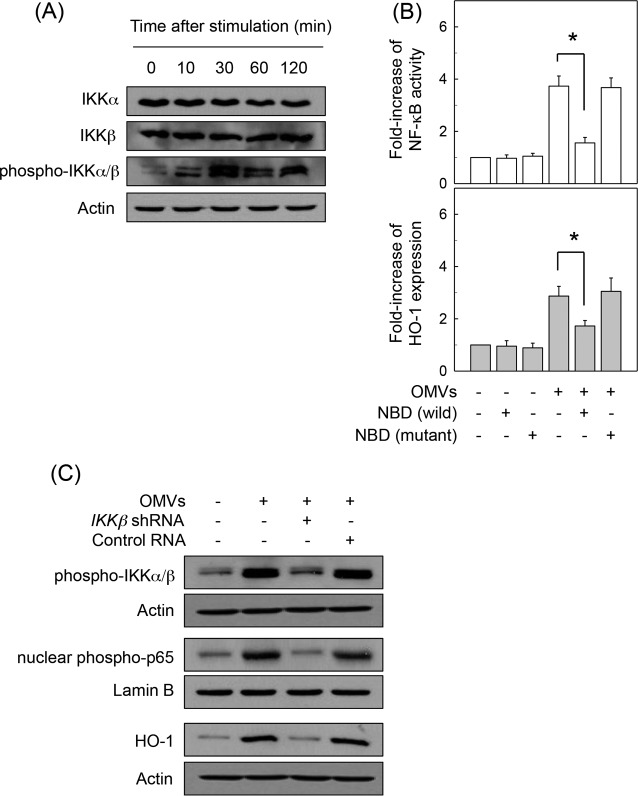
IKK activation is associated with HO-1 expression in DCs treated with crude OMVs. (A) BM-derived DCs were treated with crude OMVs (50 μg/ml) for the indicated times. Protein expression of IKKα, IKKβ, phospho-IKKα/β, and actin was assessed by immunoblot analysis. Results are representative of three independent experiments. (B) BM-derived DCs were preincubated with NBD peptide (200 μM) for 1 h, after which crude OMVs (50 μg/ml) were added for an additional 1 h (NF-κB) or 12 h (HO-1). NF-κB activity and HO-1 expression were measured by ELISA kits. Data are expressed as mean fold induction ± SEM relative to that of untreated controls (*n* = 5). *, *P* < 0.05. (C) DC2.4 cells were transfected with lentiviral vectors containing an *IKK*β shRNA or control shRNA. Transfected cells were stimulated with crude OMVs (50 μg/ml) for 1 h (phospho-IKKα/β and nuclear phospho-65) or 12 h (HO-1). Expression of each protein was analyzed by immunoblotting. Results are representative of more than three independent experiments.

We next asked whether crude OMV-induced NF-κB-dependent HO-1 expression might be associated with IKK activation in DCs. Treatment of BM-derived DCs with crude H. pylori OMVs increased phosphorylated IKKα/β expression (Fig. 3A). In this experimental system, addition of an IKK inhibitor, NBD peptide, into DCs significantly reduced the crude OMV-induced increase of NF-κB activation and HO-1 expression (Fig. 3B). To confirm these results, another experiment was performed using *IKK*β shRNA to suppress phospho-IKK expression. Transfection with lentiviruses containing *IKK*β shRNA suppressed crude OMV-induced IKK expression in DC2.4 cells (Fig. 3C, top panels). In this experimental system, the crude OMV-induced increase of phospho-p65 expression was apparently inhibited when lentiviruses containing *IKK*β shRNA were transfected (Fig. 3C, middle panels). Concurrently, *IKK*β shRNA reduced HO-1 protein expression in crude OMV-exposed cells (Fig. 3C, bottom panels). These results suggest that there is a connection between IKK–NF-κB-dependent signaling and HO-1 induction in crude OMV-exposed DCs.

**FIG 4:**
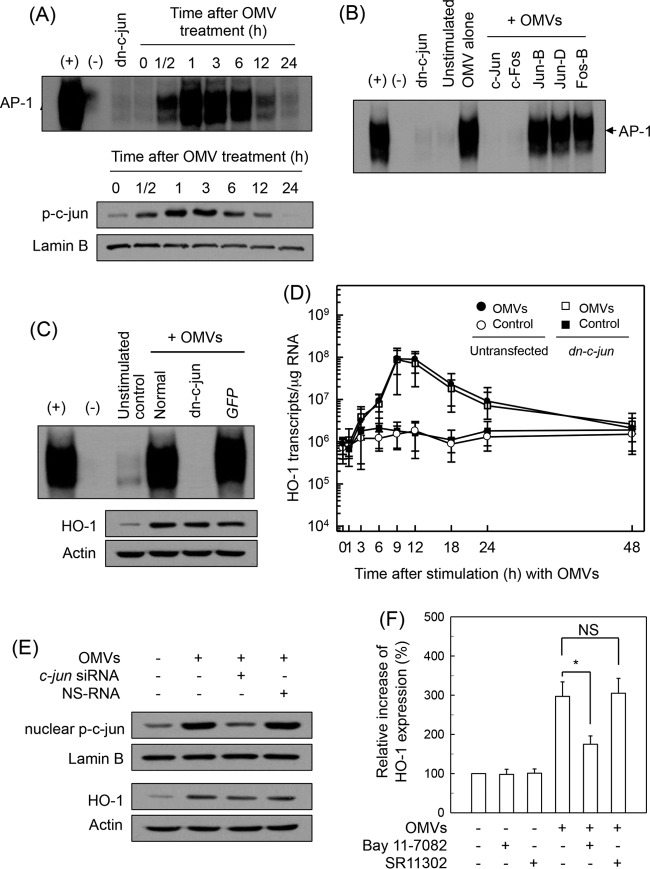
Effects of AP-1 suppression on HO-1 expression in DCs treated with crude OMVs. (A) Activation of AP-1 in DCs stimulated with crude OMVs. BM-derived DCs were treated with OMVs (50 μg/ml) for the indicated times. AP-1 DNA binding activity was assessed by EMSA. Immunoblot results for concurrent phospho-c-Jun and lamin B in nuclear extracts under the same conditions are provided beneath the EMSA panel. Results are representative of more than three independent experiments. (B) Supershift assays were performed using Abs against c-Jun, c-Fos, JunB, JunD, and FosB in BM-derived DCs stimulated with crude OMVs (50 μg/ml) for 1 h. +, positive control; −, no extracts; dn-c-jun, nuclear extracts obtained from with lentivirus-dn–*c-jun*-transfected DC2.4 cells stimulated with crude OMVs (50 μg/ml) for 1 h. (C) DC2.4 cells were transfected with either lentivirus containing dominant negative *c-jun* plasmid (dn–*c-jun*) or control virus (*GFP*). Transfected cells were stimulated with crude OMVs (50 μg/ml) for 1 h. AP-1 binding activity was assayed by EMSA. Transfected or untransfected cells were treated with OMVs (50 μg/ml) for 12 h (top panel). Expression of HO-1 and actin proteins was analyzed by immunoblotting (bottom panels). Results are representative of more than three independent experiments. (D) Transfected DC2.4 cells were treated with crude OMVs (50 μg/ml) for the indicated periods of time. Levels of HO-1 mRNA were analyzed by quantitative RT-PCR using a standard RNA. Values are expressed as means ± SD (*n* = 5). β-Actin mRNA levels in each group remained relatively constant throughout the same periods (∼ 10^6^ transcripts/μg total RNA). *, *P* < 0.05, for results compared with those in untransfected cells treated with crude OMVs. (E) DC2.4 cells were transfected with AP-1 *c-jun*-specific siRNA or nonsilencing siRNA (NS-RNA) as a control for 48 h, after which cells were combined with crude OMVs (50 μg/ml) for 1 h. Nuclear extracts were analyzed by immunoblotting with the indicated Abs (top panels). Transfected cells were stimulated with crude OMVs (50 μg/ml) for 12 h. Expression of HO-1 and actin proteins was analyzed by immunoblotting (bottom panels). Results shown are representative of more than three independent experiments. (F) Murine BM-derived DCs were preincubated with NF-κB inhibitor Bay 11-7082 (50 μM) or AP-1 inhibitor SR11302 (10 μM) for 30 min, followed by stimulation with crude OMVs (50 μg/ml) for an additional 12 h. Expression levels of HO-1 were measured by ELISA (means ± SEM; *n* = 5). *, *P* < 0.05, for results compared with those with OMV alone. NS, statistically nonsignificant.

We next determined whether AP-1 signaling might be involved in crude OMV-induced HO-1 expression. As shown in Fig. 4A, exposure of BM-derived DCs to crude OMVs increased AP-1 DNA binding, as assessed by EMSA. In addition, phosphorylated c-Jun signals were observed in OMV-exposed DCs. To further explore the effect of crude OMVs on AP-1 activation, the DNA binding activities of individual members of the AP-1 family were examined using a supershift assay. As shown in Fig. 4B, the entire AP-1 signal disappeared after treatment with Abs to c-Jun and c-Fos. The addition of Abs to JunB, JunD, or FosB did not affect the AP-1 signal induced by crude OMVs, thereby indicating that stimulation of DCs with crude OMVs may activate AP-1 consisting of c-Jun/c-Fos heterodimers.

When DC2.4 cells were infected with the lentivirus-dn–*c-jun*, OMV-induced AP-1 DNA binding activity was definitely suppressed (Fig. 4C). However, the enhanced HO-1 expression in crude OMV-treated cells did not change when AP-1 activation was suppressed. Consistent with these results, transfection with lentivirus-dn–*c-jun* did not significantly affect HO-1 mRNA expression in DC2.4 cells under crude OMV-stimulated conditions (Fig. 4D). In another experiment, *c-jun* siRNA was used to suppress AP-1 activity. The *c-jun* siRNA almost completely suppressed nuclear phospho-c-Jun expression in crude OMV-stimulated DC2.4 cells, and transfection with *c-jun* siRNA did not influence the crude OMV-induced increase of HO-1 expression in DC2.4 cells (Fig. 4E).

To confirm these results, murine BM-derived DCs were preincubated with the NF-κB inhibitor Bay 11-708 or with the AP-1 inhibitor SR11302 for 30 min and then treated with crude OMVs. As shown in Fig. 4F, crude OMVs enhanced HO-1 expression, as assessed by ELISA, and pretreatment of BM-derived DCs with Bay 11-7082 significantly attenuated crude OMV-induced HO-1 expression. However, SR11302 did not lead to a significant change of HO-1 expression in crude OMV-exposed DCs.

### Nrf2 signaling is also associated with induction of HO-1 in crude H. pylori OMV-exposed DCs.

**FIG 5:**
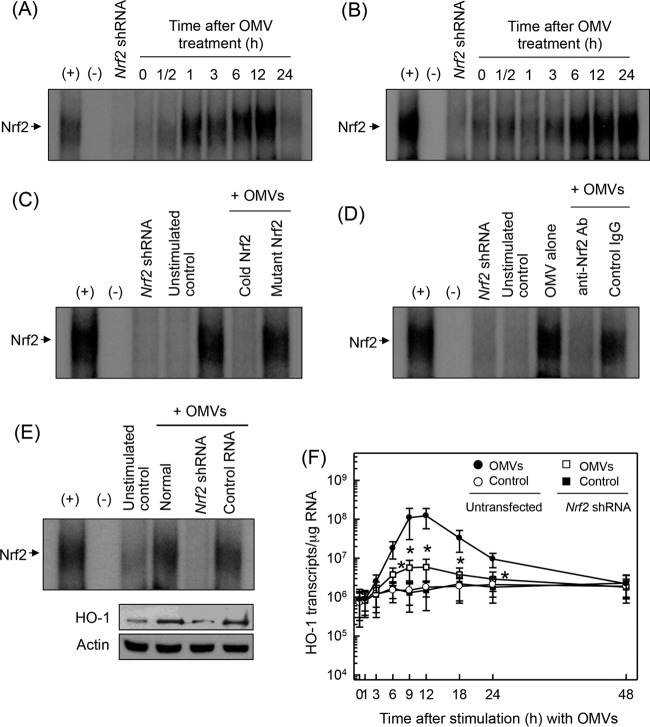
Activation of Nrf2 in DCs stimulated with crude OMVs. (A and B) Murine BM-derived DCs (A) and DC2.4 cells (B) were treated with crude OMVs (50 μg/ml) for the indicated periods of time. Nrf2 DNA binding activity was assessed by EMSA. Results are representative of more than three independent experiments. (C) Competition assays for Nrf2 signals. Murine BM-derived DCs were treated with crude OMVs (50 μg/ml) for 6 h, and nuclear extracts were then prepared. (D) Nuclear extracts were obtained from murine BM-derived DCs treated with crude OMVs (50 μg/ml) for 6 h. Supershift assays using nuclear extracts were performed using anti-Nrf2 Ab and IgG isotype control Ab. Results are representative of more than three independent experiments. +, positive control; −, no extracts; *Nrf2* shRNA, nuclear extracts obtained with *Nrf2* shRNA-transfected DC2.4 cells stimulated with crude OMVs (50 μg/ml) for 6 h. (E) DC2.4 cells were transfected with *Nrf2*-specific shRNA or a control RNA. Transfected cells were combined with crude OMVs (50 μg/ml) for 6 h. Nrf2 binding activity was assayed by EMSA (top panels). Transfected cells were treated with crude OMVs (50 μg/ml) for 12 h. Expression of HO-1 and actin proteins was analyzed by immunoblotting (bottom panels). Results are representative of more than three independent experiments. (F) Transfected cells were treated with crude OMVs (50 μg/ml) for the indicated periods of time. Levels of HO-1 mRNA were analyzed by quantitative RT-PCR using standard RNA. Values are expressed as means ± SD (*n* = 5). β-Actin mRNA levels in each group remained relatively constant throughout the same periods (∼ 10^6^ transcripts/μg total RNA). *, *P* < 0.05, for results compared with those with untransfected cells treated with OMV.

Since the promoter region of HO-1 genes contains binding sites for Nrf2 (15), we determined whether crude OMVs could activate Nrf2 in DCs. As shown in Fig. 5A, crude H. pylori OMVs increased Nrf2-DNA binding activity in murine BM-derived DCs. Similar results were observed in DC2.4 cells (Fig. 5B). To confirm the specificity of Nrf2 signals, two experiments were performed: a competition assay and a supershift assay. The addition of excess Nrf2 oligomer (cold Nrf2) to nuclear extracts obtained from crude OMV-stimulated DCs resulted in suppression of Nrf2-DNA binding activity (Fig. 5C). The supershift assay showed that Nrf2 activity apparently disappeared with the addition of Nrf2 Ab (Fig. 5D).

**FIG 6:**
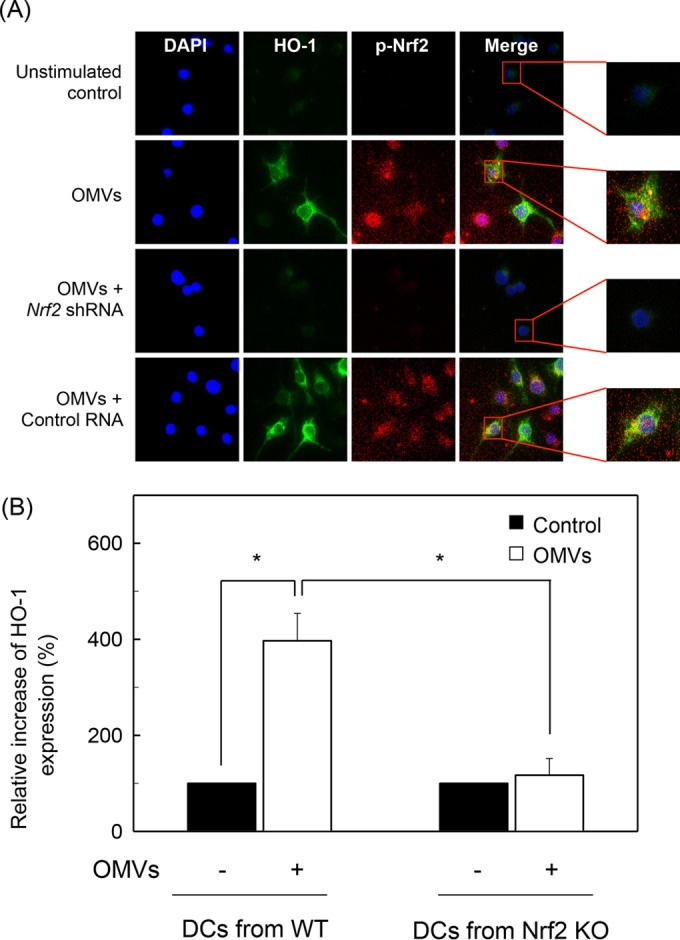
Effects of Nrf2 suppression on HO-1 expression in DCs stimulated with crude OMVs. (A) Nrf2 translocation and HO-1 expression in crude OMV-exposed DCs. DC2.4 cells were transfected with *Nrf2*-specific shRNA or a control RNA. Transfected cells were treated with crude OMVs (50 μg/ml) for 6 h, and immunofluorescence microscopy was performed. Each group of cells was stained with anti-HO-1 Ab (green), anti-Nrf2 Ab (red), and 4′,6′-diamidino-2-phenylindole (DAPI; blue, nucleus). Data are representative of at least five experiments. (B) HO-1 expression in DCs derived from wild-type and Nrf2^−/−^ knockout mice. DCs derived from wild-type (WT) or Nrf2^−/−^ knockout (KO) mice were stimulated with crude OMVs (50 μg/ml) for 12 h. Expression of HO-1 proteins in each panel was measured by ELISA (means ± SEM; *n* = 5). *, *P* < 0.01.

We next asked whether HO-1 induction was associated with Nrf2 activation in crude OMV-exposed cells. Transfection with lentivirus containing *Nrf2* shRNA almost completely suppressed Nrf2 activity in DC2.4 cells stimulated with crude OMVs (Fig. 5E). In this experimental system, a significant difference in HO-1 protein expression was observed between cells transfected with *Nrf2* shRNA and untransfected cells under crude OMV-treated conditions. To confirm this result, cells transfected with *Nrf2* shRNA were stimulated with crude OMVs. The level of HO-1 mRNA was then determined by quantitative RT-PCR. Transfection with *Nrf2* shRNA significantly suppressed HO-1 mRNA expression in DC2.4 cells compared with that in untransfected cells under crude OMV-treated conditions (Fig. 5F). Another experiment was performed using immunofluorescence microscopy. Experiments showed that phospho-Nrf2 and HO-1 signals increased in crude OMV-exposed cells, in which *Nrf2* shRNA transfection definitely reduced the extent of phospho-Nrf2 and HO-1 expression (Fig. 6A). To confirm these results, an experimental model of Nrf2^−/−^ knockout mice was used. As shown in Fig. 6B, crude OMVs increased the expression of HO-1 in DCs derived from wild-type mice. In addition, there was a significant difference in HO-1 expression levels between DCs derived from wild-type mice and those derived from Nrf2^−/−^ knockout mice. These results indicate that Nrf2 signaling also plays an important role in the induction of HO-1 in crude OMV-stimulated DCs.

### Akt/mTOR pathway is involved in HO-1 expression induced by crude H. pylori OMV stimulation.

**FIG 7:**
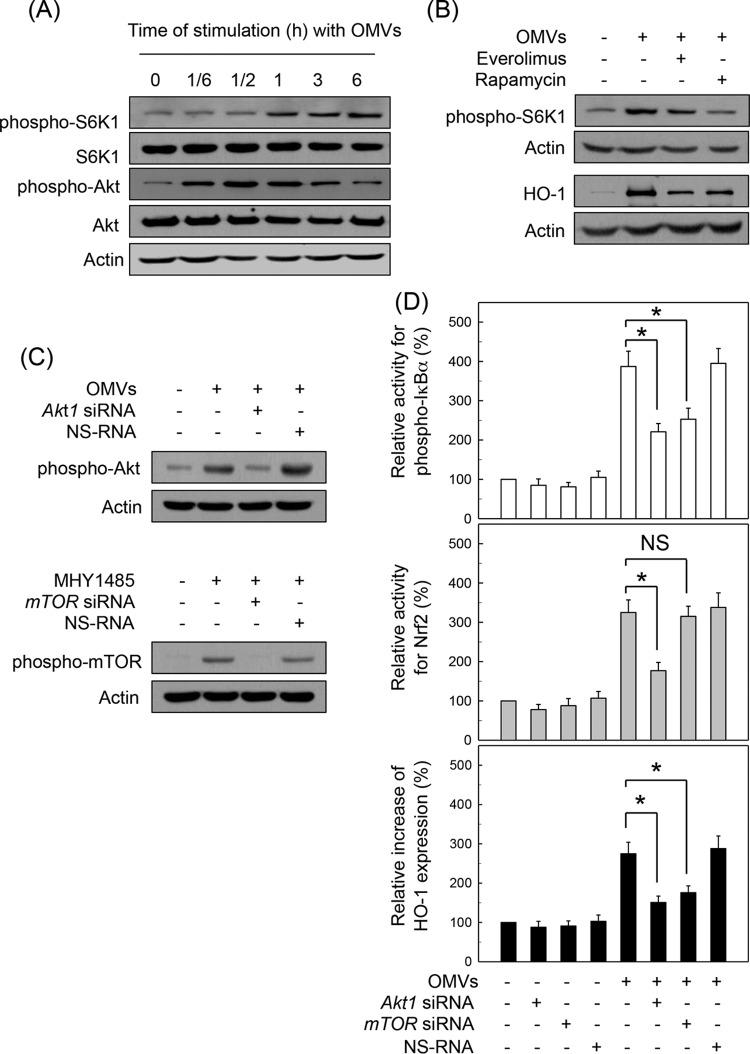
Akt/mTOR pathway is involved in HO-1 expression induced by H. pylori OMV stimulation. (A) Murine BM-derived DCs were stimulated with crude OMVs (50 μg/ml) for the indicated time periods. Expression levels of phospho-S6K1, S6K1, phospho-Akt, Akt, and actin were measured by immunoblot analysis. Results are representative of more than three independent experiments. (B) DCs were pretreated with everolimus (1 μM) or rapamycin (100 nM) for 30 min, after which cells were stimulated with crude OMVs (50 μg/ml) for another 1 h (phospho-S6K1) or 12 h (HO-1). Protein levels of phospho-S6K1, HO-1, and actin in total cell extracts were determined using immunoblot analysis. Results are representative of more than five independent experiments. (C) DC2.4 cells were transfected with *Akt1*- or *mTOR*-specific silencing siRNA (siRNA) or nonsilencing control siRNA (NS RNA) for 48 h. Transfected cells were stimulated with crude OMVs (50 μg/ml) or the mTOR inducer MHY1485 (5 μM) for another 1 h, after which expression of phospho-Akt and phospho-mTOR was analyzed by immunoblotting. Results shown are representative of three independent experiments. (D) Transfected cells were either left untreated or stimulated with crude OMVs (50 μg/ml) for another 1 h (phospho-IκBα), 6 h (Nrf2), or 12 h (HO-1). Each ELISA kit measured activities of phospho-IκBα and Nrf2, as well as HO-1 expression. Data are expressed as mean fold induction ± SEM (%) relative to that in untreated controls (*n* = 5). *, *P* < 0.05.

mTOR activation is regulated by various upstream signaling molecules including Akt (26). mTOR regulates mRNA translation by affecting the phosphorylation or activity of several translation factors such as S6K1. We determined whether crude H. pylori OMV-induced HO-1 upregulation is associated with Akt-mTOR signaling. As shown in Fig. 7A, treatment of murine BM-derived DCs with crude OMVs increased expression of phospho-S6K1 and phospho-Akt. In this experimental system, pretreatment with mTOR inhibitors such as everolimus and rapamycin resulted in definite decreases of phospho-S6K1 expression compared to treatment with OMV alone (Fig. 7B, top panels). Pretreatment with everolimus or rapamycin also inhibited crude OMV-induced HO-1 protein expression in murine BM-derived DCs (Fig. 7B, bottom panels). In another experiment, transfection with siRNA against *Akt1* or *mTOR* was used to suppress the activities of Akt or mTOR signals in DC2.4 cells (Fig. 7C). In this experimental system, transfection with siRNA against *Akt1* clearly resulted in inhibition of phospho-IκBα and Nrf2 and expression of HO-1 protein under a stimulated condition with crude OMVs (Fig. 7D). In addition, transfection with *mTOR* siRNA significantly inhibited the increase in crude OMV-induced phospho-IκBα and HO-1 expression. However, there was no significant change in Nrf2 activity between untransfected and *mTOR* siRNA-transfected cells under crude OMV-stimulated conditions.

**FIG 8:**
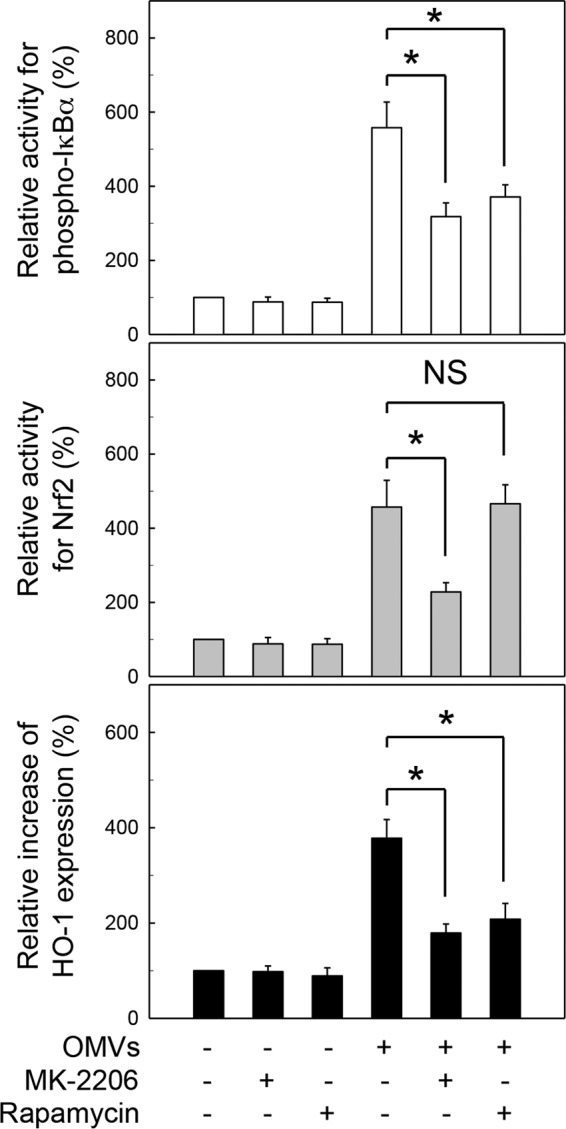
Relationship between suppression of Akt-mTOR activity and HO-1 expression in human PBMC-derived DCs stimulated with crude H. pylori OMVs. Human PBMC-derived DCs were pretreated with MK-2206 (100 nM) or rapamycin (100 nM) for 30 min, after which cells were stimulated with crude OMVs (50 μg/ml) for another 1 h (phospho-IκBα), 6 h (Nrf2), or 12 h (HO-1). Activities of phospho-IκBα and Nrf2 and expression of HO-1 were measured by each ELISA kit. Data are expressed as mean fold induction ± SEM (%) relative to that in untreated controls (*n* = 5). *, *P* < 0.01; NS, not significant.

To confirm these results, a model with human monocyte-derived DCs was used. As shown in Fig. 8, exposure of human monocyte-derived DCs to crude OMVs significantly enhanced the activities of phospho-IκBα and Nrf2, as well as HO-1 expression, compared to levels in an unstimulated control. In this experimental system, pretreatment with MK-2206 (Akt inhibitor) significantly attenuated the activities of phospho-IκBα and Nrf2 and expression of HO-1 under OMV-exposed conditions. There was no significant change in Nrf2 activity between rapamycin (mTOR inhibitor) pretreatment and control cells stimulated with crude OMVs. However, rapamycin significantly inhibited the increased activity of phospho-IκBα and the increased expression of HO-1 in crude OMV-exposed cells.

## DISCUSSION

The majority of H. pylori in the stomach remains unattached to the surface epithelium and releases OMVs even though bacteria can adhere to gastric epithelial cells (5, 6). The gastric mucosa lacks the organized and diffuse lymphoid tissues found in the gut (4). However, follicular DCs are identified in H. pylori-associated gastric mucosa-associated lymphoid tissue (MALT) lymphomas, in which antigens of H. pylori are found in follicular DCs (4, 27). Therefore, it is reasonable to assume that crude H. pylori OMVs can closely contact and be exposed to DCs at sites of H. pylori infection in the stomach. In experiments reported here, we demonstrate that the exposure of DCs to H. pylori-derived crude OMVs upregulated expression of HO-1 at the mRNA and protein levels.

H. pylori OMVs contain many surface elements and bacterial proteins, including CagA, VacA, and LPS (7–11). In the present study, crude OMVs obtained from a CagA-negative isogenic mutant strain induced less HO-1 expression than crude OMVs obtained from a wild-type strain. Tanaka H et al. demonstrated that translocation of CagA protein into DCs and its tyrosine-phosphorylation were observed during wild-type H. pylori infection but not during *cagA*-negative H. pylori infection, suggesting that the tyrosine phosphorylation of CagA is caused in DCs as well as gastric epithelial cells (28). Our results are consistent with findings that the expression of HO-1 increased in gastric mononuclear cells of human patients and macrophages of mice infected with *cagA*^+^ H. pylori strains (16). Considering that HO-1 inhibited CagA phosphorylation in gastric epithelial cells (29), HO-1 expression induced by OMVs may be involved in control of gastric inflammation.

Transcription factors such as NF-κB, AP-1, and Nrf2 regulate a variety of inflammatory responses (30–32). The promoter region of HO-1 genes contains binding sites for these transcription factors. The present study demonstrated that signals of NF-κB, AP-1, and Nrf2 were activated by DC exposure to crude OMVs. Many papers have demonstrated that HO-1 expression is regulated by Nrf2 signaling in response to stimuli (33–35). However, it remains controversial whether HO-1 induction in DCs is associated with NF-κB, AP-1, or Nrf2. In the present study, suppression of NF-κB activity either by transfection with lentivirus–IκBα-AA and *p65* siRNA or by pretreatment with the chemical inhibitor Bay 11-7082 significantly reduced H. pylori OMV-induced HO-1 expression in DCs. In addition, crude OMV-induced activation of Nrf2 played a critical role in the upregulation of HO-1 in DCs. These results were confirmed by experiments using DCs isolated from Nrf2^−/−^ knockout mice. However, suppression of AP-1 signals did not result in a significant change in HO-1 expression. Therefore, both NF-κB- and Nrf2-dependent expression of HO-1 may be a distinctive signature of DCs exposure to crude H. pylori OMVs.

Akt signaling is an important event underlying HO-1 expression (36). Although H. pylori can activate Akt and mTOR molecules in gastric epithelial cells (37), there is no report regarding H. pylori OMV-induced Akt and mTOR signaling in DCs. Moreover, cooperation between NF-κB and Akt-mTOR signaling or Nrf2 and Akt-mTOR signaling is not clear in OMV-exposed cells. To gain insight into the signaling pathways involved in H. pylori OMV-induced HO-1 upregulation, we investigated whether Akt–mTOR–IKK–NF-κB and Akt-mTOR-Nrf2 signaling might be related to HO-1 expression in DCs. In the present study, treatment of DCs with crude OMVs increased expression of phospho-Akt and phospho-S6K1. Suppression of phospho-Akt in OMV-exposed DCs significantly attenuated activation of both NF-κB and Nrf2. In addition, Akt suppression was associated with inhibition of crude OMV-induced HO-1 expression. However, suppression of mTOR activity significantly inhibited IKK activity and HO-1 expression in crude OMV-exposed DCs but not Nrf2 activity. These results were confirmed using human monocyte-derived DCs. These results suggest that two differential pathways are involved in the induction of HO-1 in response to stimulation with crude H. pylori OMVs. The exposure of DCs to crude OMVs activates two signaling cascades involving Akt–mTOR–IKK–NF-κB and Akt-Nrf2, leading to HO-1 induction.

LPS markedly downregulated HO-1 gene expression in primary human DCs via upregulated expression of Bach1, a critical transcriptional repressor of HO-1 (38). In contrast to the level in primary human mononuclear cells, HO-1 was upregulated in LPS-treated murine macrophages (38). These findings suggest the existence of species-specific differences between murine and human DCs with respect to HO-1 induction. In the present study, enhanced expression of HO-1 was noted in human PBMC-derived DCs and murine BM-derived DCs exposed to crude OMVs. Therefore, upregulation of HO-1 by H. pylori-derived OMVs does not seem to be a species-specific difference.

H. pylori produces small numbers of OMVs during the logarithmic growth phase and large amounts of OMVs during stationary phase (10). Based on this finding, the present study used a 72-h liquid culture to obtain large amounts of OMVs. However, the crude OMVs are likely to contain contaminants, such as broken flagella and proteins from lysed bacterial cells. Therefore, experiments using pure H. pylori OMVs without any contaminants are necessary.

HO-1 expression is downregulated by maturation stimuli, indicating that HO-1 expression inhibits DC maturation (21). Consequently, induction of HO-1 led to decreased levels of reactive oxygen species (ROS) following stimulation of DCs with LPS. ROS levels were inversely correlated with the degree of DC maturation (39), and antioxidants blocked DC maturation (40). These findings suggest that the decrease in ROS levels by HO-1 may be involved in inhibiting DC maturation (21). Because H. pylori inhibits DC maturation (41–43), upregulation of HO-1 by H. pylori OMVs is likely associated with the maintenance of an immature state in DCs. Nevertheless, further studies are required to clarify the maturation process in H. pylori OMV-exposed DCs.

**FIG 9:**
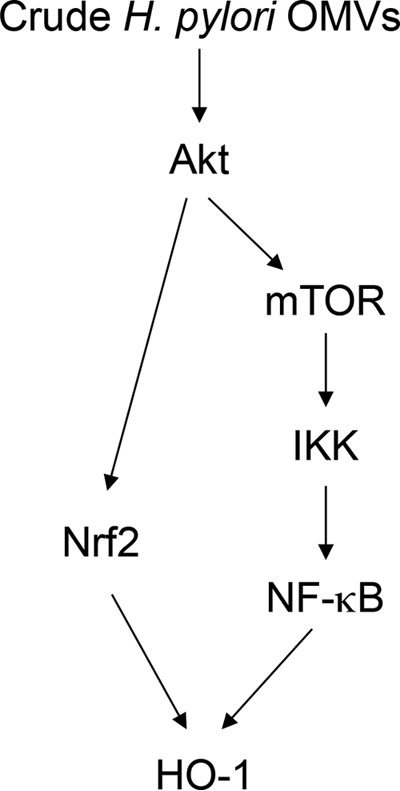
Schematic summary indicating how signal transduction by crude H. pylori OMVs induces HO-1 expression in DCs.

In summary, H. pylori-derived crude OMVs upregulate the expression of HO-1 in DCs. Activation of two signaling pathways, Akt–mTOR–IKK–NF-κB and Akt-Nrf2, may play important roles in this process (Fig. 9). These results suggest that increased HO-1 expression in DCs modulates inflammatory responses in H. pylori infection.
